# Efficacy of Vector Guard®, a mosaic alpha-cypermethrin and piperonyl butoxide-treated net, for the control of pyrethroid-resistant malaria vectors: a non-inferiority experimental hut evaluation in Benin

**DOI:** 10.1186/s13071-025-07038-w

**Published:** 2025-10-06

**Authors:** Judicael Nounagnon, Martial Gbegbo, Abel Agbevo, Estelle Vigninou, Boris N’dombidjé, Corine Ngufor

**Affiliations:** 1 Centre de Recherches Entomologiques de Cotonou (CREC), Cotonou, Benin; 2 Pan-African Malaria Vector Research Consortium (PAMVERC), Cotonou, Benin; 3 African Institute for Research in Infectious Diseases (AIRID), Cotonou, Benin; 4 London School of Hygiene and Tropical Medicine (LSHTM), London, WC1E 7HT UK

**Keywords:** Insecticide-treated nets, Vector Guard, Olyset Plus, Royal Sentry, Piperonyl butoxide

## Abstract

**Background:**

Insecticide-treated nets (ITNs) incorporating pyrethroids with piperonyl butoxide (PBO) have demonstrated enhanced effectiveness against malaria transmitted by pyrethroid-resistant vectors compared with standard pyrethroid-only ITNs. To sustain progress in malaria prevention, a broader portfolio of effective pyrethroid-PBO nets is required to promote market diversity and strengthen supply resilience. In this study, we evaluated the entomological efficacy and wash durability of Vector Guard®, a novel mosaic alpha-cypermethrin–PBO ITN, against pyrethroid-resistant *Anopheles gambiae* sensu lato (*An. gambiae* s.l.) in southern Benin.

**Methods:**

An experimental hut trial was conducted in Covè, Benin, against wild, free-flying *An. gambiae* s.l. The effectiveness of the Vector Guard® ITN was tested unwashed and after 20 standardized washes, and also compared to two WHO-prequalified ITNs: Olyset® Plus (a permethrin-PBO net) and Royal Sentry® 2.0 (an alpha-cypermethrin-only net). Primary outcomes were mosquito mortality and blood-feeding protection. Susceptibility bioassays were conducted to assess local vector resistance to insecticides. Laboratory cone and tunnel tests were also performed to help explain the finding in the experimental huts. Chemical content analyses were performed to investigate active ingredient wash retention. Vector Guard® was assessed for its non-inferiority to Olyset® Plus following WHO guidance.

**Results:**

The wild *An. gambiae* s.l. population at Covè exhibited high frequencies of pyrethroid resistance, with PBO pre-exposure restoring partial susceptibility to alpha-cypermethrin (34% vs 4% mortality) but not to permethrin (2.0% vs 2.1% mortality). A total of 6799 females were collected in the experimental huts. Vector Guard® outperformed both Royal Sentry® 2.0 and Olyset® Plus across all entomological endpoints. Mortality with Vector Guard® was significantly higher than with Olyset® Plus both when unwashed (36.4% vs 17.5%, *p* < 0.001) and after 20 washes (17.2% vs 8.7%, *p* < 0.001). Non-inferiority analysis with pooled data for unwashed and washed nets confirmed that Vector Guard® was non-inferior to Olyset® Plus in terms of both mortality (odds ratio [OR] 2.71, 95% confidence interval [CI] 2.26–3.24, non-inferiority margin [NIM] 0.423) and blood-feeding protection (OR 0.53, 95% CI 0.45–0.62, NIM: 1.359). These findings were supported by the results from the cone and tunnel tests. Chemical analysis showed higher wash retention of active ingredients in Vector Guard® (83% for PBO and > 94% for alpha-cypermethrin) compared to Olyset® Plus (40.2% for PBO and 69.6% for permethrin).

**Conclusions:**

Vector Guard® demonstrated superior entomological efficacy and wash durability compared to Royal Sentry® 2.0 and Olyset® Plus, and fulfilled WHO non-inferiority criteria for mosquito mortality and blood-feeding inhibition. These findings support its addition to the WHO list of prequalified pyrethroid-PBO ITNs and its potential to provide improved malaria control when deployed on a large scale in areas with high levels of pyrethroid resistance.

**Graphical Abstract:**

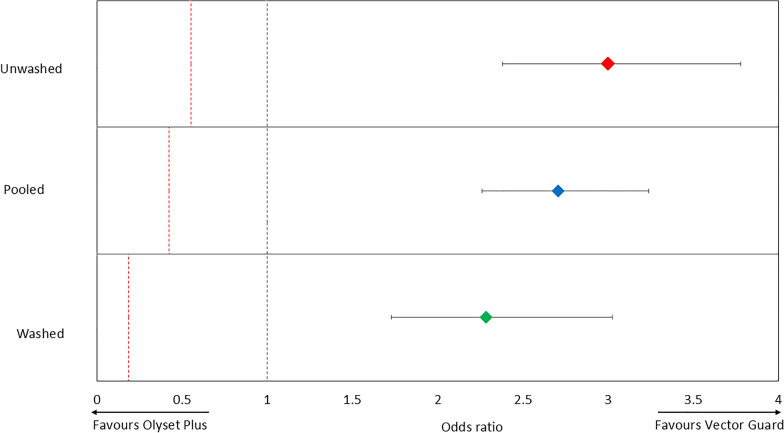

## Background

Insecticide-treated nets (ITNs) remain the cornerstone of malaria prevention in endemic regions and have contributed substantially to the marked reductions in malaria incidence and mortality over the past two decades [1]. However, the rapid spread of pyrethroid resistance among major malaria vectors has undermined their effectiveness [2]. In response, a new generation of ITNs has been developed which combines pyrethroids with additional active ingredients, such as synergists or alternative insecticide classes—to enhance efficacy against pyrethroid-resistant mosquito populations [3].

A key innovation has been the incorporation of piperonyl butoxide (PBO), a synergist that inhibits oxidases responsible for metabolic resistance to pyrethroids [4]. Pyrethroid-PBO nets have shown superior efficacy against pyrethroid-resistant vectors in both experimental and operational settings. Multiple experimental hut trials across Africa have confirmed their advantage over pyrethroid-only nets [4, 5], and two large-scale cluster-randomized controlled trials (cRCTs) in Tanzania and Uganda demonstrated significant reductions in malaria incidence and parasite prevalence in children using pyrethroid-PBO nets compared with standard ITNs [6, 7]. These findings have established pyrethroid-PBO nets as an essential tool for malaria control in areas with documented pyrethroid resistance [8].

Consequently, several pyrethroid-PBO ITN brands have been prequalified by the WHO and adopted by National Malaria Control Programs (NMCPs) across sub-Saharan Africa [3, 9]. Expanding the diversity of available pyrethroid-PBO nets remains important to strengthen market competition, meet procurement demands and ensure reliable supply. Within this context, Vector Guard®, a new alpha-cypermethrin–PBO net developed by Disease Control Technologies LLC (Greer, SC, USA) represents a promising addition to the next-generation ITN portfolio. The net features a mosaic design, with the roof panel incorporating both alpha-cypermethrin and PBO, while the side panels contain alpha-cypermethrin alone. In line with WHO guidance, Vector Guard® must demonstrate entomological efficacy superior to pyrethroid-only nets and non-inferiority to a WHO-prequalified pyrethroid-PBO net with proven public health value to qualify for prequalification and programmatic use [10].

Here, we report the findings of an experimental hut trial conducted in Covè, southern Benin that evaluated the efficacy and wash resistance of Vector Guard® against wild, free-flying, pyrethroid-resistant *Anopheles gambiae* sensu lato (*An. gambiae* s.l.). Nets were tested both unwashed and after 20 standardized washes, and compared against two WHO-prequalified ITNs: Olyset® Plus (Sumitomo Chemical Co., Ltd, Japan), a permethrin-PBO net with demonstrated public health value in cRCTs [7], and Royal Sentry® (Disease Control Technologies), a standard alpha-cypermethrin-only ITN. The trial followed WHO guidelines for ITN evaluation [10], and outcomes were analyzed using WHO-defined criteria for non-inferiority and superiority in entomological endpoints, namely mosquito mortality and blood-feeding inhibition [11].

## Methods

### Experimental hut site and vector profile

The study was conducted at the Centre de Recherches Entomologiques de Cotonou/London School of Hygiene & Tropical Medicine (CREC/LSHTM) experimental hut station in Covè, southern Benin (7.21°N, 2.34°E), situated within a rice-growing area that provides favorable conditions for mosquito breeding. The local vector population consists of a mixture of *Anopheles coluzzii* and *Anopheles gambiae* sensu stricto (*An. gambiae* s.s.), with *An. gambiae *s.s. accounting for approximately 23% of the mosquito population, particularly during the dry season. The vector population is characterized by intense pyrethroid resistance, with WHO susceptibility bioassays showing > 90% survival following exposure to alpha-cypermethrin, deltamethrin and permethrin [12, 13]. Resistance is primarily mediated by a very high frequency (> 90%) of the L1014F knockdown resistance (*kdr*) mutation, together with over-expression of metabolic detoxification enzymes [5, 14].

The trial was implemented using seven West African–style experimental huts.

### Susceptibility bioassays

To assess the frequency of pyrethroid resistance in the wild mosquito population during the hut trial, WHO susceptibility bioassays were performed using 2- to 5-day-old, unfed adult F1 female mosquitoes reared from larvae collected around the experimental huts [15].

Four replicates of 20–25 mosquitoes were exposed to filter papers impregnated with the WHO discriminating concentrations of alpha-cypermethrin (0.05%) and permethrin (0.75%), alongside untreated controls. The susceptible *An. gambiae* Kisumu strain was tested in parallel as a reference. Knockdown was recorded after 60 min, and mortality after a 24-h holding period.

To investigate the role of metabolic resistance mechanisms, particularly cytochrome P450 monooxygenases (P450), synergist bioassays with PBO (4%) were conducted. Eight replicates of 25 mosquitoes were first exposed to PBO-treated filter papers for 60 min; of these, four replicates were subsequently exposed to alpha-cypermethrin (0.05%) and the remaining four to permethrin (0.75%) for a further 60 min. An additional four replicates were exposed to PBO alone to assess its independent effects, while untreated papers served as negative controls.

### Experimental hut treatments

Vector Guard® is manufactured from high-density polyethylene (HDPE) with a 120-denier roof panel incorporating a mixture of alpha-cypermethrin (5.8 g/kg ± 25%) and PBO (23.2 g/kg ± 25%). The side panels are treated with alpha-cypermethrin alone at the same target dose (5.8 g/kg ± 25%).

The candidate net was evaluated against two WHO-prequalified ITNs: (i) Olyset® Plus: a monofilament HDPE net made with 150-denier yarn, incorporating permethrin (20 g/kg) and PBO (10 g/kg); and (ii) Royal Sentry® 2.0: a 120-denier HDPE net treated with alpha-cypermethrin (5.8 g/kg ± 25%).

All three net types were evaluated in both the unwashed and washed (20 times [20×]) conditions following WHO standard procedures [10]. In total, seven treatments were assessed in the experimental hut trial: (i) untreated polyethylene net (control); (ii) Royal Sentry® 2.0 (alpha-cypermethrin-only), unwashed; (iii) Royal Sentry® 2.0 (alpha-cypermethrin-only) washed 20×; (iv) Olyset® Plus (permethrin + PBO), unwashed; (v) Olyset® Plus (permethrin + PBO), washed 20×; (vi) Vector Guard® (alpha-cypermethrin + PBO) unwashed; (vii) Vector Guard® (alpha-cypermethrin + PBO), washed 20×.

To simulate wear and tear, all nets—including untreated controls—were deliberately holed in accordance with WHO guidelines, resulting in six 4× 4-cm holes (2 holes on each long side and 1 hole on each short side). Net washing procedures also followed WHO standard protocols. Each net was washed in an aluminum bowl containing 10 l of water with 2 g/l of Savon de Marseille (a traditional hard soap made from vegetable oils), agitated for a total of 10 min. Nets were then rinsed in clean water using the same method, dried horizontally in the shade and stored at ambient temperature between washes. The washing intervals were set at 2 days for Vector Guard® and Olyset® Plus, and 1 day for Royal Sentry® 2.0, in line with regeneration studies.

### Hut trial design

To minimize potential bias due to hut position, treatments were rotated weekly across the seven experimental huts using a randomized Latin Square Design to reduce carry-over effects. The hut trial was conducted over 42 nights between February and April 2022. Data collection occurred over six consecutive nights each week, with the seventh day reserved for cleaning and airing the huts in preparation for the next rotation. Six replicate nets were tested per treatment, with nets rotated daily within each week. Each night, from 21:00 hour to 06:00 hour, seven consenting human volunteers slept in the huts to attract wild, free-flying mosquitoes. Each morning, volunteers collected mosquitoes from the hut compartments (under the net, in the room and in the veranda) using a torch and aspirator and placed them in labeled plastic cups. Collections were transferred to the field laboratory for morphological identification and assessed for immediate mortality and blood-feeding status. Surviving female *An. gambiae* s.l. were maintained at 27 ± 2 °C and 75 ± 10% relative humidity with access to 10% glucose solution, and delayed mortality was recorded after 24 h.

The efficacy of treatments in the experimental huts was evaluated using the following outcome measures:

- Entry rate: total number of mosquitoes collected per hut
- Deterrence (%): reduction in mosquito entry in treated huts compared to the untreated control
- Exophily (%): proportion of mosquitoes found in the veranda, indicating treatment-induced exiting behavior
- Inside net (%): proportion of mosquitoes collected inside the net
- Blood-feeding rate (%): proportion of mosquitoes that were blood-fed
- Blood-feeding inhibition (%): reduction in blood-feeding in treated huts relative to the control
- Personal protection (%): reduction in the number of blood-fed mosquitoes in the treated hut compared to the control
- Mortality (%): proportion of mosquitoes that died within 24 h post-collection.

### Supplementary cone and tunnel tests

To supplement hut trial data, laboratory cone bioassays and tunnel tests were conducted to assess the bioavailability and potency of active ingredients in each ITN. These bioassays followed existing WHO guidelines [16–18]. Net samples were taken from both unwashed and washed nets (20×) of each ITN type. Cone bioassays were performed using the pyrethroid-susceptible *An. gambiae* s.s. Kisumu strain to evaluate the efficacy of the pyrethroid component, while tunnel tests used the pyrethroid-resistant *An. gambiae* s.l. Covè strain to assess the added effect of PBO in Vector Guard® and Olyset® Plus ITNs. All assays were conducted under controlled conditions (27 ± 2 °C and 75 ± 10% relative humidity).

In the cone bioassays, 8–12 unfed, 2- to 5-day-old *An. gambiae* s.s. Kisumu mosquitoes were exposed in two batches of 4–6 per cone for 3 min on each piece of ITN. After exposure, mosquitoes were transferred to labeled holding cups, provided with 10% glucose solution and observed for knockdown at 60 min and mortality at 24 h.

Tunnel tests were conducted on two randomly selected net pieces from each ITN type and wash status. For Vector Guard®, only roof panel samples were tested. The tunnel apparatus simulates natural host-seeking behavior and consists of a glass chamber divided into two sections by a wooden frame holding the net sample. A guinea pig bait was placed in a cage at one end of the tunnel, and approximately 100 unfed, 5- to 8-day-old *An. gambiae* s.l. Covè mosquitoes were released at dusk into the opposite end. Net samples were perforated with nine 1-cm-diameter holes to allow mosquito passage. The following morning, mosquitoes were collected, and immediate mortality and blood-feeding status were recorded. Surviving mosquitoes were held in labeled cups with access to 10% glucose solution and monitored for delayed mortality at 24 h.

### Chemical analysis of insecticide content

To assess within- and between-net variation in active ingredient content, as well as wash-resistance, chemical analyses were conducted on Vector Guard® net samples by the CRA-W reference laboratory (Walloon Agricultural Research Center, Gembloux, Belgium). Net pieces (30 × 30 cm) taken from the experimental hut trial were analyzed for alpha-cypermethrin and PBO content using Collaborative International Pesticides Analytical Council (CIPAC) methods 54/LN/M/3.2, 454/LN/M3/3 and 33/LN/(M)/3. These methods involve extraction of the active ingredients in a water bath at 85–90 °C for 45 min using heptane and dicyclohexyl phthalate as the internal standard, followed by quantification via gas chromatography with flame ionization detection (GC-FID). The identity of the active ingredients was confirmed through comparison with authentic standards. Each net sample was analyzed individually, and average concentrations were calculated per treatment group.

### Statistical analysis

Proportional outcomes, including mosquito mortality, blood-feeding and exophily, were compared across treatments using logistic regression, while numerical outcomes such as mosquito entry were analyzed using negative binomial regression. Each outcome was modeled separately, with adjustments for variation between huts, sleepers and trial days included as fixed effects [14].

Non-inferiority analyses comparing Vector Guard® to Olyset® Plus were performed in line with WHO guidelines [15]. Vector Guard® was considered to be non-inferior in terms of mosquito mortality if the lower bound of the 95% confidence interval (CI) for the odds ratio (OR) exceeded the non-inferiority margin (NIM), and in terms of blood-feeding if the upper bound of the 95% CI was below the NIM. The NIM was calculated to reflect a 7% difference in efficacy (mortality or blood-feeding) of Vector Guard® relative to Olyset® Plus.

Superiority of Vector Guard® over Royal Sentry® 2.0 was assessed based on significantly higher mosquito mortality and lower blood-feeding rates at the 5% significance level (*p* < 0.05). Analyses were conducted for unwashed and washed nets separately, as well as pooled, to assess overall product efficacy across the net’s lifespan. All statistical analyses were performed using Stata version 18 (StataCorp, College Station, TX, USA).

### Compliance with Organization for Economic Co-operation and Development principles of Good Laboratory Practice

To ensure compliance with the Organization for Economic Co-operation and Development (OECD) Good Laboratory Practice (GLP) principles, all phases of the study—from protocol development to reporting—were conducted under strict quality control. Equipment was calibrated, ITNs were verified for expiry and certification and mosquito strains were handled according to standard operating practices. The candidate net was sourced from three production batches and stored under monitored conditions. Validated systems were used for data collection and processing, and all procedures were documented. The quality assurance team at CREC/LSHTM inspected all critical phases and found no non-conformances. External GLP inspections by the South African National Accreditation System (SANAS) in 2022 also reported full compliance.

## Results

### WHO cylinder bioassay results

The frequency of resistance to pyrethroids was very high in wild *An. gambiae* s.l. from the Covè hut site, with mortality rates of only 2.1% with permethrin 0.75% and 4% with alpha-cypermethrin 0.05% (Table 1). Pre-exposure to PBO followed by exposure to alpha-cypermethrin increased mortality substantially to 34%, indicating partial restoration of susceptibility. In contrast, pre-exposure to PBO had no impact on mortality when combined with exposure to permethrin, which remained low at 2%. PBO alone and control treatments resulted in negligible mortality (0–3.1%). In comparison, both permethrin and alpha-cypermethrin induced 100% mortality against the susceptible *An. gambiae* Kisumu strain, confirming full susceptibility.

**Table 1:** WHO susceptibility cylinder bioassay results with wild mosquitoes collected as larvae from the experimental hut station during the trial

Mosquito strain	Insecticide	Mosquitoes exposed (*N*)	Knockdown at 60 min (*N*)	Knockdown (%)	95% CI	Dead mosquitoes (*N*)	Mortality (%)	95% CI
Pyrethroid-resistant *Anopheles gambiae* s.l. Covè	Control	97	1	1	0–3	3	3.1	0–7
	Permethrin 0.75%	94	0	0	–	2	2.1	0–5
	Alpha-cypermethrin 0.05%	99	0	0	–	4	4	0–8
	PBO 4%	98	0	0	–	0	0	0
	PBO + permethrin	98	0	0	–	2	2	0–5
	PBO + alphacypermethrin	94	28	29.8	21–39	32	34	24–44
Susceptible *Anopheles gambiae* Kisumu	Control	101	0	0	–	2	1.98	0–5
	Permethrin 0.75%	100	100	100	95-100	100	100	95-100
	Alpha-cypermethrin 0.05%	100	100	100	95-100	100	100	95-100

*CI* Confidence interval, *PBO* piperonyl butoxide,* s.l.* sensu lato

#### Experimental hut results

##### Entry and exiting results

A total of 6799 female *An. gambiae* s.l. were collected in the experimental huts in Covè during the study period. The average number of mosquitoes caught per night across treatments ranged from 20 to 26 (Table 2). Vector Guard® induced the strongest deterrent effect relative to the control, both when unwashed (21.1%) and after 20 washes (12.5%). In contrast, Royal Sentry® 2.0 showed minimal deterrence, particularly after 20 washes (1.5%). The proportion of mosquitoes exiting the hut was significantly higher in all insecticide-treated arms compared to the untreated control (37.9%). Among unwashed nets, exit rates were comparable, with 60.5% exit rate for Royal Sentry® 2.0, 64.8% for Olyset® Plus and 65.3% for Vector Guard®. After 20 washes, Vector Guard® maintained a high exit rate (65.3% vs 62.8%; *p* = 0.348), whereas exiting decreased more noticeably with Royal Sentry® 2.0 (54.6%) and Olyset® Plus (50.1%), suggesting that in comparison to these latter two ITNs, excito-repellent activity of the Vector Guard® ITN was better retained after washing (*p* < 0.05).

**Table 2:** Entry/exiting of wild, free-flying, pyrethroid-resistant *Anopheles gambiae *sensu lato into/from experimental huts in Covè, southern Benin

Entry/exiting results	Net type						
	Control	Royal Sentry® 2.0		Olyset® Plus		Vector Guard®	
	–	Unwashed	Washed 20×	Unwashed	Washed 20×	Unwashed	Washed 20×
Total* N* females caught	1055^a,b^	936^c,d^	1039^a^	957^b,c^	1083^a^	832^d^	923^c,d^
Average catch per night (*N*)	25	22	25	23	26	20	22
Deterrence (%)	–	11.2	1.5	9.2	0.0	21.1	12.5
Total* N* exiting mosquitoes	400	567	568	621	543	544	580
Exiting mosquitoes (%)	37.9^a^	60.5^b^	54.6^c^	64.8^b^	50.1^c^	65.3^b^	62.8^b^
95% CI	34.9–40.8	57.4–63.7	51.6–57.6	61.8–67.9	47.1–53.1	62.1–68.6	59.7–65.9

Values in the same row followed by the same superscript letter do not differ significantly at the 5% level according to the logistic regression analysis

*CI *Confidence interval

##### Blood-feeding results

**Fig. 1:**
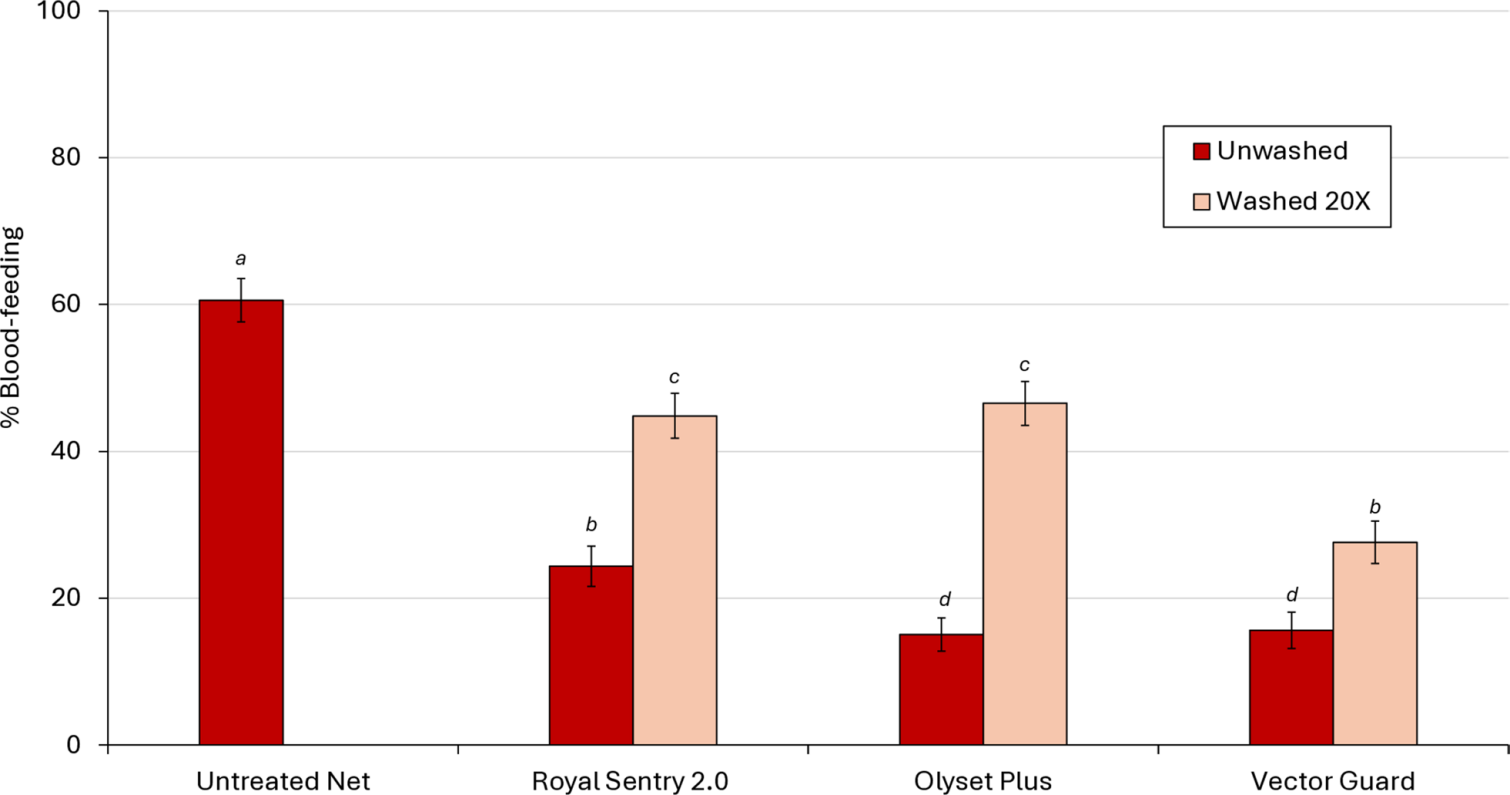
Blood-feeding of wild, free-flying pyrethroid-resistant *Anopheles gambiae* entering experimental huts in Covè, southern Benin. Bars with the same lowercase letter label are not significantly different at *p* > 0.05 according to logistic regression. Error bars represent the 95% confidence interval

The blood-feeding rate with the control (untreated net) was 60.6% (Fig. 1; Table 3). Among unwashed nets, Olyset® Plus and Vector Guard® achieved the lowest blood-feeding rates at 15.0% and 15.6%, respectively, both significantly lower than that of Royal Sentry® 2.0 (24.3%; *p* < 0.05). After 20 washes, Vector Guard® maintained superior its performance, with a blood-feeding rate of 27.6%, significantly lower than that of Olyset® Plus (46.5%; *p* < 0.001) and Royal Sentry® 2.0 (44.8%; *p* < 0.001).

**Table 3:** Blood-feeding of wild, free-flying, pyrethroid-resistant *Anopheles gambiae *sensu lato entering experimental huts in Covè, southern Benin

Blood-feeding parameters	Net type						
	Control	Royal Sentry 2.0®		Olyset® Plus		Vector Guard®	
	–	Unwashed	Washed 20×	Unwashed	Washed 20×	Unwashed	Washed 20×
Total* N* females caught	1055	936	1039	957	1083	832	923
Total* N* blood-fed mosquitoes	639	228	466	144	504	130	255
Blood-feeding (%)	60.5^a^	24.3^b^	44.8^c^	15.0^d^	46.5^c^	15.6^d^	27.6^b^
95% CI	57.6–63.5	21.6–27.1	41.8–47.9	12.8–17.3	43.6–49.5	13.2–18.1	24.7–30.5
Blood-feeding inhibition (%)	–	59.7	25.9	75.1	23.1	74.2	54.3
95% CI	–	56.6–62.9	23.9–28.6	72.4–77.9	20.7–25.7	71.2–77.2	51.2–57.6
Personal protection (%)	–	64.3	27.0	77.4	21.1	79.6	60.0

Values in the same row followed by the same superscript letter do not differ significantly at the 5% level according to the logistic regression analysis

*CI* Confidence interval

Blood-feeding inhibition mirrored these trends: Vector Guard® and Olyset® Plus achieved 74.2% and 75.1% inhibition, respectively, when unwashed, compared to 59.7% with Royal Sentry® 2.0 (*p* < 0.001). After 20 washes, blood-feeding inhibition with Vector Guard® remained high at 54.3%, which is significantly greater than that of Olyset® Plus (23.1%) and Royal Sentry® 2.0 (25.9%; *p* < 0.001 for both). In addition, personal protection levels with unwashed nets were highest with Vector Guard® (79.7%) and Olyset® Plus (77.5%) while Royal Sentry® 2.0 provided the lowest level of personal protection (64.3%). After 20 washes, the personal protection level remained high with Vector Guard® (60.0%) while this declined substantially with both ITN types Olyset® Plus (21.1%) and Royal Sentry® 2.0 (27.1%). These findings highlight the superior and greater wash-resistant blood-feeding inhibition and personal protection provided by Vector Guard® against pyrethroid-resistant mosquitoes.

##### Mortality results

**Fig. 2:**
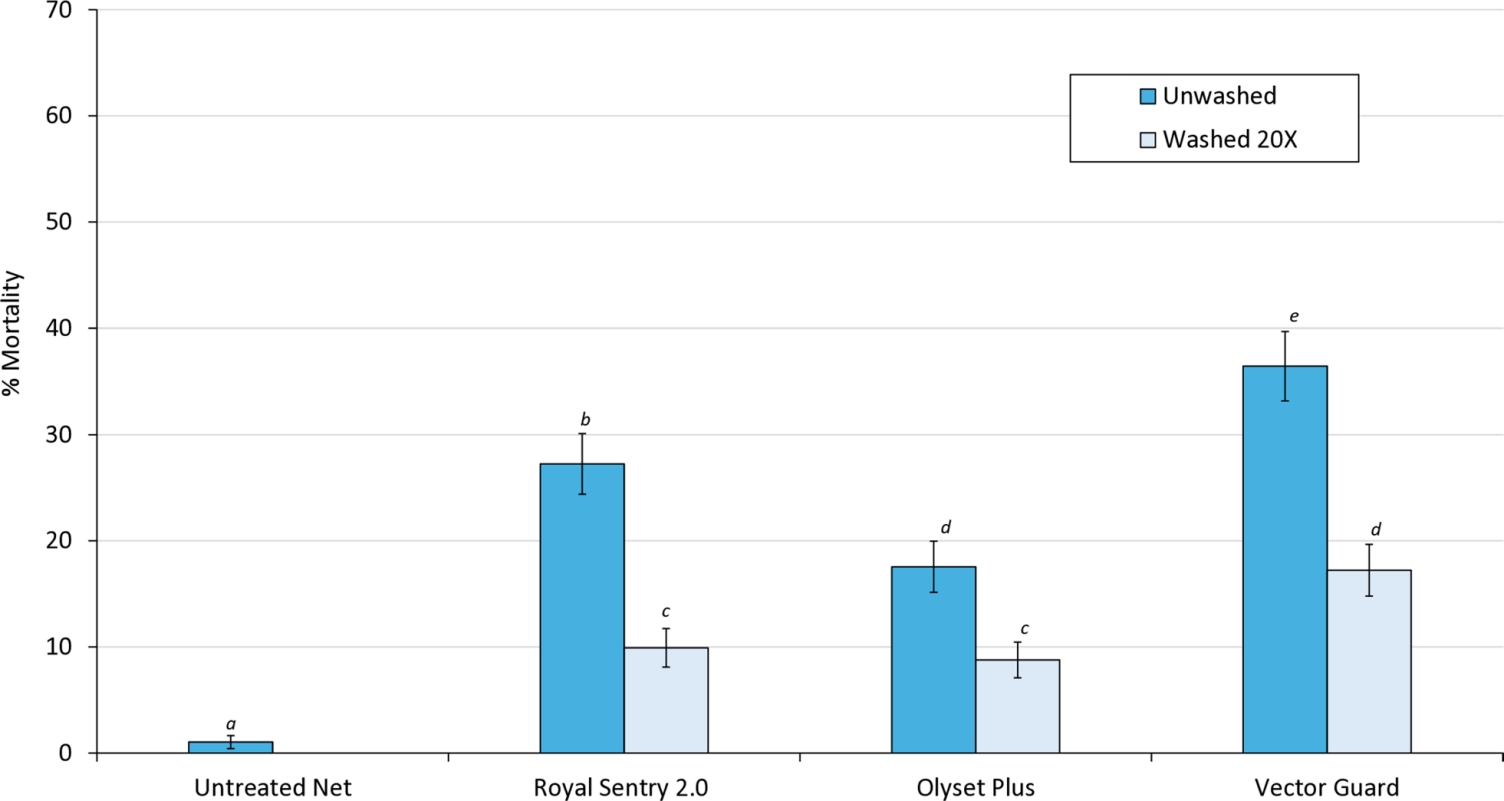
Mortality (at 24 h) of wild, free-flying, pyrethroid-resistant *Anopheles gambiae *sensu lato entering experimental huts in Covè, southern Benin. Bars with the same lowercase letter label are not significantly different at *p* > 0.05 according to logistic regression. Error bars represent the 95% confidence interval

The mortality of wild pyrethroid-resistant *An. gambiae* s.l. with the control net was 1% (Fig. 2; Table 4). With unwashed nets, the highest mortality rate was achieved with Vector Guard® (36.4%) compared to Olyset® Plus (17.6%; *p* < 0.001) and Royal Sentry® 2.0 (27.2%; *p* < 0.001). A similar trend was observed with nets washed 20×; mortality was significantly higher with Vector Guard® compared to Olyset® Plus (17.2% vs 8.8%; *p* < 0.001) and Royal Sentry® 2.0 (17.2% vs 9.9%; *p* < 0.001).

**Table 4:** Mortality of wild, free-flying, pyrethroid-resistant *Anopheles gambiae* sensu lato entering experimental huts in Covè, southern Benin

Mortality parameters	Net type						
	Control	Royal Sentry 2.0		Olyset® Plus		Vector Guard®	
	–	Unwashed	Washed 20×	Unwashed	Washed 20×	Unwashed	Washed 20×
Total* N* females caught	1055	936	1039	957	1083	832	923
Mosquitoes dead after 24 h (*N*)	11	255	103	168	95	303	159
Mosquitoes dead after 24 h (%)	1.0^a^	27.2^b^	9.9^c^	17.5^d^	8.7^c^	36.4^e^	17.2^d^
95% CI	0.4–1.7	(24.4–30.1)	(8.1–11.7)	(15.1–19.9)	(7.1–10.5)	(33.2–39.7)	(14.8–19.7)

Values in the same row followed by the same superscript letter do not differ significantly at the 5% level according to the logistic regression analysis

*CI* Confidence interval

#### Non-inferiority assessment of vector Guard® to Olyset® plus

##### Mortality outcomes

**Fig. 3:**
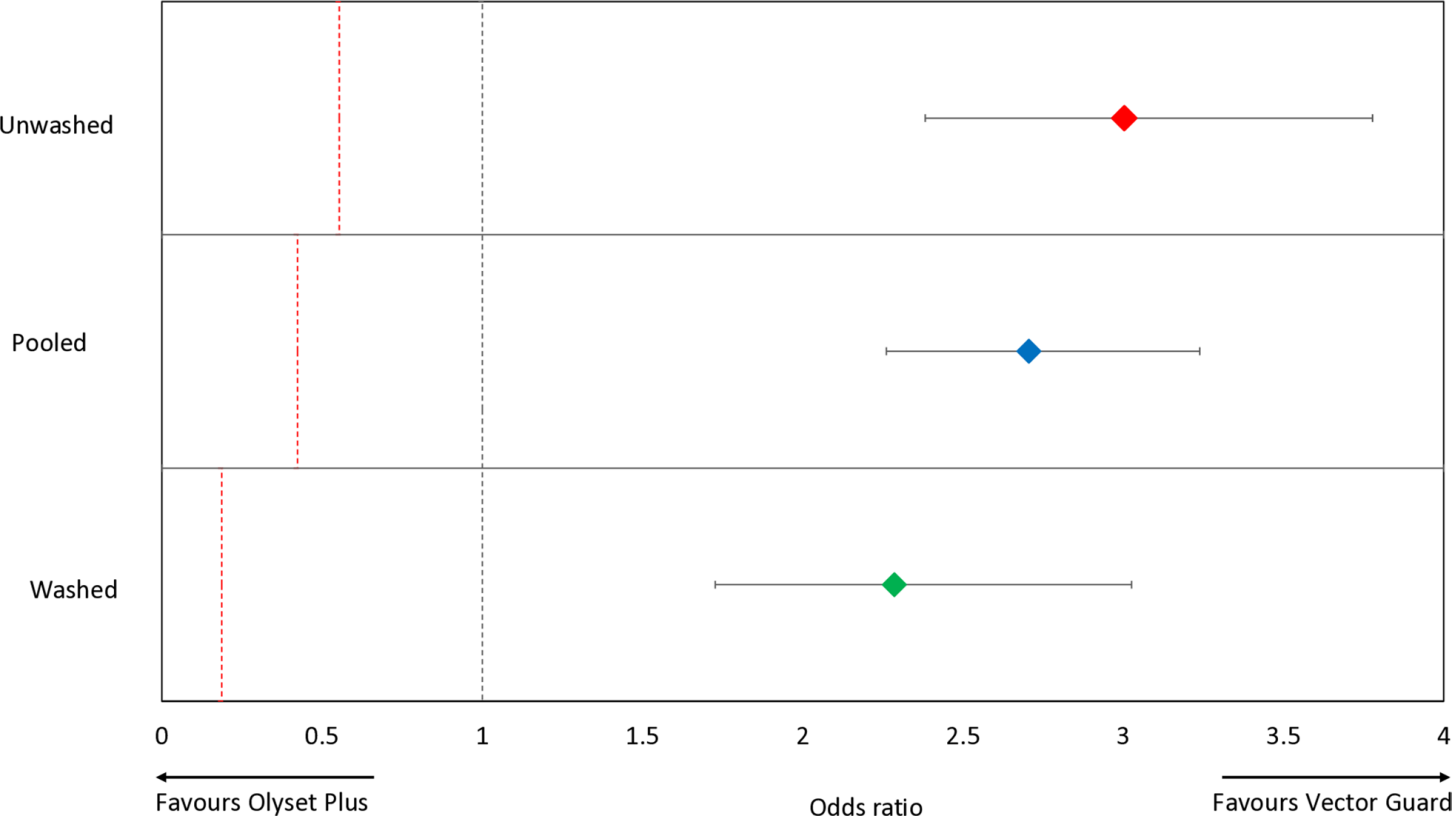
Odds ratios describing the difference in mosquito mortality after 24 h between the candidate net (Vector Guard®) and the active comparator (Olyset® Plus). Error bars represent 95% confidence intervals. The black dashed line represents an odds ratio of 1, indicating no difference between the candidate net and the active comparator. The red dashed line represents the non-inferiority margin. The candidate net is considered to be non-inferior to the active comparator if the lower 95% confidence interval of the odds ratio is higher than the non-inferiority margin

 Vector Guard® met WHO non-inferiority criteria for mosquito mortality when compared to Olyset® Plus under both unwashed and washed conditions (Fig. 3; Table 5). When unwashed, Vector Guard® induced significantly higher mortality (36.4%) than Olyset® Plus (17.5%; *p* < 0.001), with an OR of 3.003 (95% CI 2.384–3.784). The lower bound of the confidence interval exceeded the non-inferiority margin (NIM 0.554), indicating both non-inferiority and superiority. After 20 washes, mortality remained significantly higher with Vector Guard® (17.2%) compared to Olyset® Plus (8.7%; *p* < 0.001), with an OR of 2.286 (95% CI 1.727–3.026) and a corresponding NIM of 0.187. As the lower confidence limit was also well above the NIM, Vector Guard® was confirmed to be non-inferior and also superior to Olyset® Plus after 20 washes. When the results were pooled across washing conditions, Vector Guard® maintained a higher overall mortality rate (26.3%) than Olyset® Plus (12.8%), with an OR of 2.706 (95% CI 2.261–3.239) and a NIM of 0.423, again confirming non-inferiority and superiority. Vector Guard® also consistently outperformed Royal Sentry® in terms of vector mortality (*p* < 0.001).

**Table 5:** Non-inferiority and superiority analyses comparing the effect of Vector Guard® to Olyset® Plus and Royal Sentry 2.0 in terms of mosquito mortality and blood-feeding outcomes in experimental huts

	Unwashed		20× Washed		Pooled	
Non-inferiority assessment^a^ (Vector Guard® vs Olyset Plus®)						
Analyses	Odds ratio (95% CI)	NIM	Odds ratio (95% CI)	NIM	Odds ratio (95% CI)	NIM
Mortality	3.003	0.554	2.286	0.187	2.706	0.423
	(2.382–3.784)	Non-inferior	(1.727–3.026)	Non-inferior	(2.261–3.239)	Non-inferior
Blood-feeding	1.001	1.596	0.38	1.323	0.531	1.359
	(0.767–1.306)	Non-Inferior	(0.312–0.463)	Non-Inferior	(0.453–0.622)	Non-Inferior
Superiority assessment^a^ (Vector Guard® vs Royal Sentry®)						
Analyses	Odds ratio (95% CI)	*p*-Value	Odds ratio (95% CI)	*p*-Value	Odds ratio (95% CI)	*p*-Value
Mortality	1.563	< 0.001	2.08	< 0.001	1.741	< 0.001
	(1.252–1.951)	Superior	(1.576–2.741)	Superior	(1.467–2.066)	Superior
Blood-feeding	0.61	< 0.001	0.424	< 0.001	0.477	< 0.001
	(0.475–0.783)	Superior	(0.347–0.518)	Superior	(0.408–0.559)	Superior

*CI *Confidence interval,* NIM* non-inferiority margin

^a^To fulfill non-inferiority criteria, the lower bound of the 95% CI of the odds ratio must exceed NIM for mortality while the upper bound of the 95% CI of the odds ratio must not exceed NIM for blood-feeding. Vector Guard® must also be superior to Royal Sentry® 2.0 at the 5% level

##### Blood-feeding outcomes

**Fig. 4:**
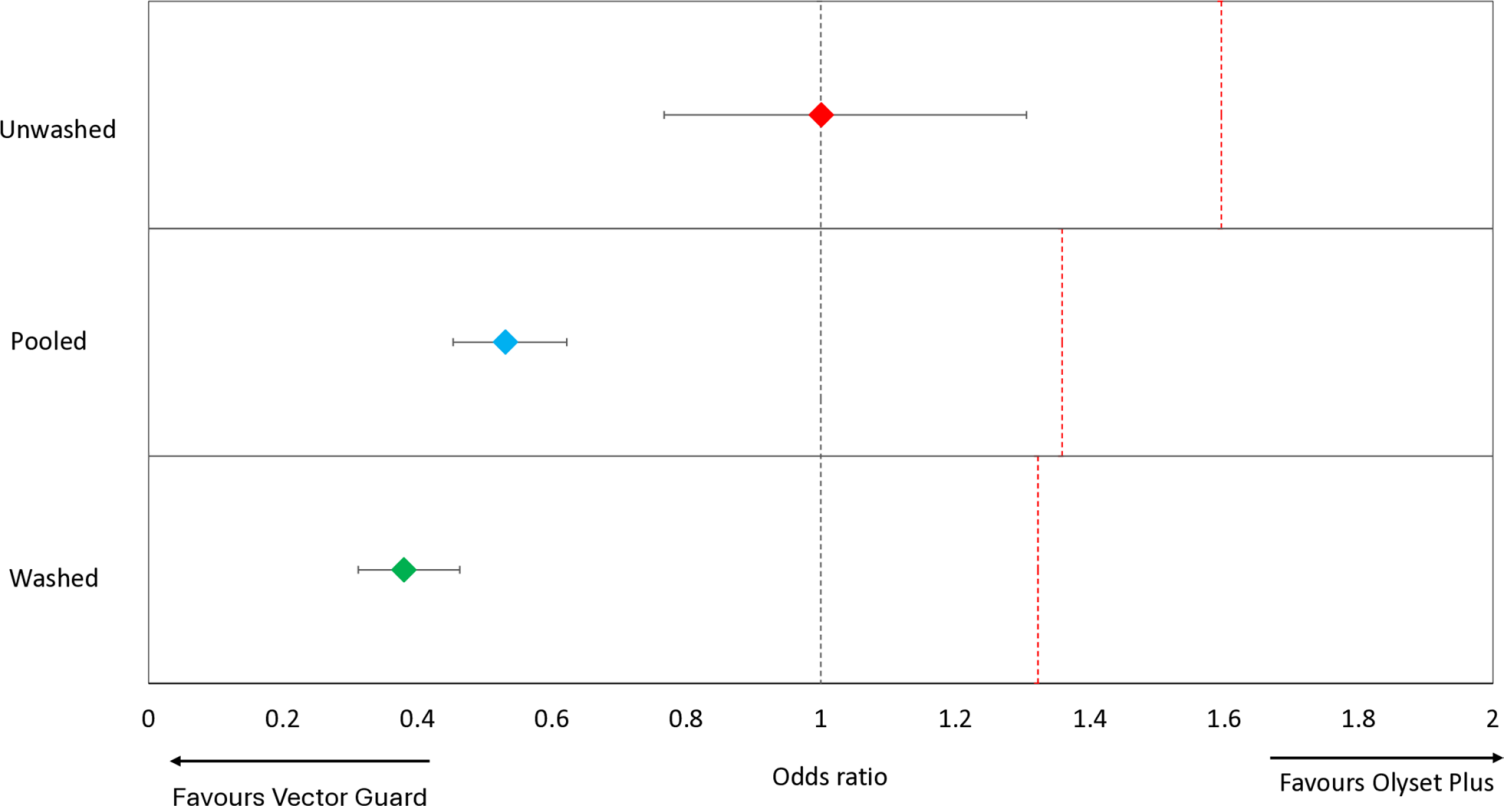
Odds ratios describing the difference in mosquito blood-feeding between the candidate net (Vector Guard®) and the active comparator (Olyset® Plus). Error bars represent 95% confidence intervals. The black dashed line represents the odds ratio of 1, indicating no difference between the candidate net and the active comparator. The red dashed line represents the non-inferiority margin. The candidate net is considered to be non-inferior to the active comparator if the lower 95% confidence interval of the odds ratio is higher than the non-inferiority margin

 In terms of blood-feeding inhibition, Vector Guard® was non-inferior to Olyset® Plus under all conditions (Fig. 4, Table 5). When unwashed, the blood-feeding rate was 15.6% for Vector Guard® and 15.0% for Olyset® Plus (OR 1.001, 95% CI 0.767–1.306), with the upper confidence limit well below the NIM of 1.596, confirming non-inferiority. After 20 washes, blood-feeding remained significantly lower with Vector Guard® (27.6%) compared to Olyset® Plus (46.5%), with an OR of 0.39 (95% CI 0.312–0.463) and a NIM of 1.323. The upper bound of the confidence interval was well below the NIM, supporting both non-inferiority and superiority. When pooled across washing conditions, Vector Guard® also demonstrated lower blood-feeding rates (21.9%) than Olyset® Plus (31.7%), with an OR of 0.531 (95% CI 0.453–0.622) and a NIM of 1.359, confirming sustained non-inferiority and superior performance. In all conditions tested, Vector Guard® also showed greater blood-feeding inhibition than Royal Sentry® (*p* < 0.001).

### Supplementary laboratory bioassay results

#### Cone bioassay results

**Fig. 5:**
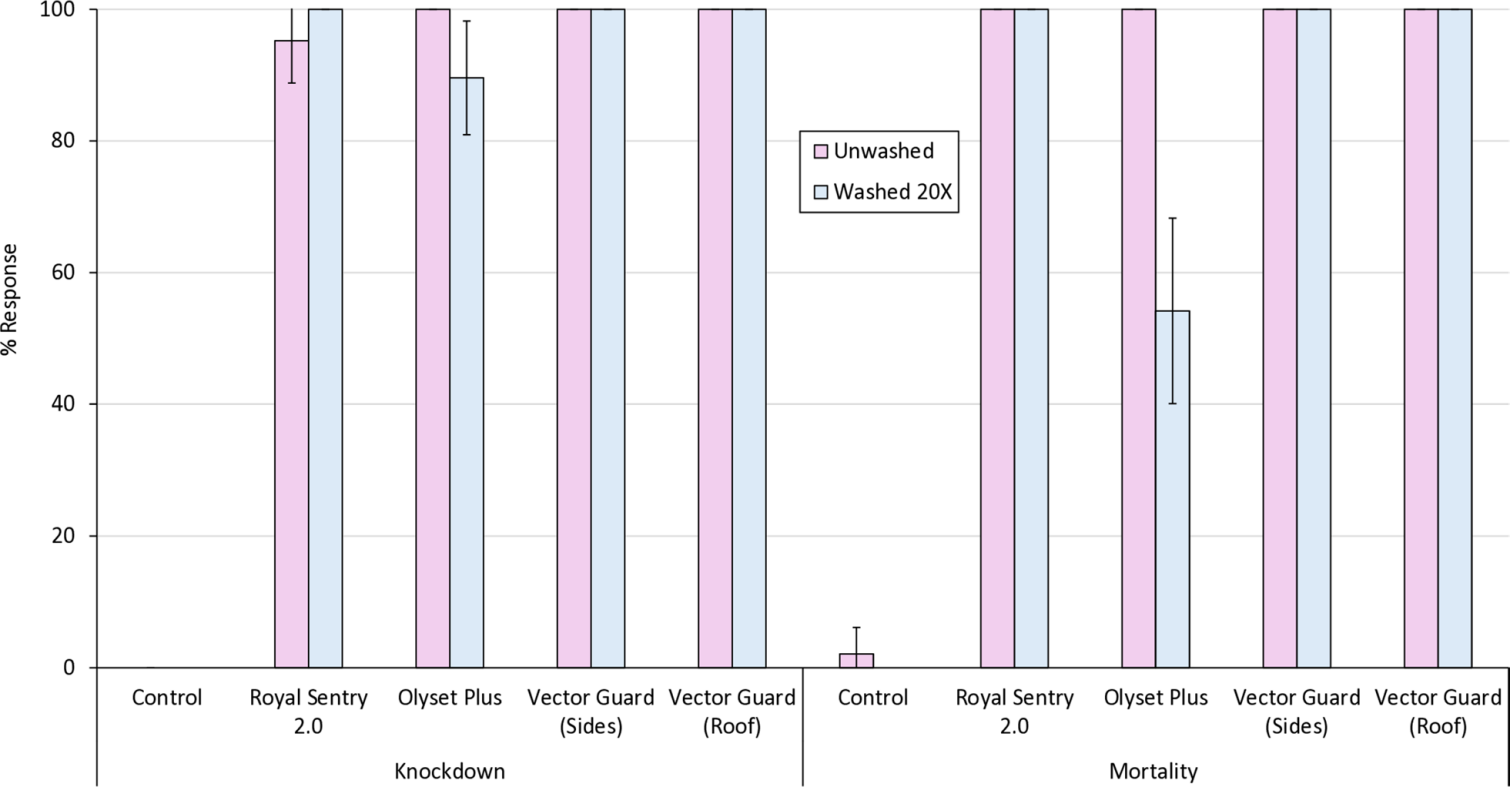
Knockdown and mortality (24 h) of susceptible *Anopheles gambiae* sensu stricto Kisumu strain in supplementary cone bioassays. Approximately 8–12 mosquitoes were exposed to each of the 5 net pieces cut from unwashed and washed nets before and after the hut trial for 3 min in two batches of 4–6. Error bars represent 95% confidence intervals

 In the cone bioassays with the susceptible *Anopheles gambiae* s.s. Kisumu strain, all ITNs—including Vector Guard®, Olyset® Plus and Royal Sentry® 2.0—achieved high knockdown and mortality rates when unwashed (Fig. 5). After 20 washes, Vector Guard® (both side and roof panels) and Royal Sentry® 2.0 maintained high mortality rates of > 80%, while Olyset® Plus exhibited a substantial decline, with mortality dropping to around 60%.

#### Tunnel test results

 In tunnel tests against the pyrethroid-resistant *Anopheles gambiae* s.l. Covè strain, Vector Guard® demonstrated the highest efficacy among the ITNs tested (Table 6). When unwashed, Vector Guard® (roof panels) induced 99% mortality, outperforming Olyset® Plus (91.9%) and Royal Sentry® 2.0 (76.4%). Following 20 washes, mortality with Vector Guard® remained high at 72.1%, substantially higher than that of Olyset® Plus, which dropped markedly to 20.8%, while Royal Sentry® 2.0 maintained 77.4%. Blood-feeding inhibition remained > 90% for all three nets when unwashed, but after 20 washes, Vector Guard® continued to provide strong blood-feeding protection (93.8% blood-feeding inhibition), comparable to Royal Sentry® 2.0 (95.5% blood-feeding inhibition), whereas Olyset® Plus experienced a sharp decline in blood-feeding inhibition to 53.7%.

**Table 6:** Summary results of tunnel tests with pyrethroid-resistant *Anopheles gambiae* sensu lato Covè strain

Wash status	Treatments	Exposed mosquitoes (*N*)	Dead mosquitoes (*N*)	Mortality, % (95% CI)	Blood-fed mosquitoes (*N*)	Blood-fed mosquitoes, % (95% CI)	Blood-feeding inhibition (*%*)
Unwashed	Control	173	2	1.2 (0–2.7)	113	65.3 (58.2–72.4)	–
	Royal Sentry® 2.0	203	155	76.4 (70.5–82.2)	12	5.9 (2.7–9.2)	90.95
	Olyset® Plus Net	184	169	91.9 (87.9–95.8)	2	1.1 (0–2.6)	98.3
	Vector Guard® (roof)	208	206	99 (97.7–100)	0	0	100
Washed 20×	Royal Sentry® 2.0	239	185	77.4 (72.1–82.7)	7	2.9 (0.8–5.1)	95.52
	Olyset® Plus Net	192	40	20.8 (15.1–26.6)	58	30.2 (23.7–36.7)	53.7
	Vector Guard® (roof)	197	142	72.1 (65.8–78.3)	8	4.1 (1.3–6.8)	93.78

*CI* Confidence interval

### Chemical analysis of net pieces

The active ingredient content in all unwashed ITNs was within the defined specifications declared by the manufacturers. Retention of PBO after 20 washes was lowest with Olyset® Plus (40.2%) (Table 7). Side panels of the Vector Guard® ITN showed high wash-retention of alpha-cypermethrin (94.5%). Between the pyrethroid-PBO ITNs, Vector Guard® (roof) showed higher levels of wash-retention of both active ingredients (94.5% for alpha-cypermethrin and 83.4% for PBO) compared to Olyset® Plus (69.6% for permethrin and 40.2% for PBO). The wash-resistance index of PBO was thus higher with Vector Guard® (99.1%) than with Olyset® Plus (95.5%).

**Table 7:** Chemical content of unwashed and washed net pieces taken before and after the experimental hut trial in Covè, Benin

ITN type	Active ingredient	Active ingredient content (g/kg)		Active ingredient retention (%)
		Unwashed	Washed 20×	
Royal Sentry® 2.0	Alpha-cypermethrin	5.3	4.7	88.7
Olyset® Plus	Permethrin	18.4	12.8	69.6
	PBO	8.7	3.5	40.2
Vector Guard®	Alpha-cypermethrin (roof)	5.5	5.2	94.5
	PBO (roof)	16.3	13.6	83.4
	Alpha-cypermethrin (side)	5.3	5.0	94.3

*ITN* Insecticide-treated net,* PBO* piperonyl butoxide

## Discussion

This experimental hut study provides robust entomological evidence on the efficacy and wash durability of Vector Guard®, a new mosaic ITN combining alpha-cypermethrin and PBO, against wild, free-flying, pyrethroid-resistant *An. gambiae* s.l. in southern Benin. Vector Guard® demonstrated superior and more durable protection than Royal Sentry® 2.0, a standard pyrethroid-only ITN, and was non-inferior and, in many outcomes, superior to Olyset® Plus, a WHO-prequalified pyrethroid-PBO net with documented epidemiological impact [7]. Across all major entomological endpoints, including mosquito deterrence, exiting behavior, blood-feeding inhibition and mortality, Vector Guard® consistently outperformed both comparator nets. Crucially, these effects were sustained after 20 standardized washes, underscoring the net’s wash durability and its potential to provide longer-lasting protection under field conditions.

The superior efficacy of Vector Guard® over Royal Sentry® 2.0 observed in the present study aligns with findings from multiple experimental hut and community trials across sub-Saharan Africa comparing pyrethroid-PBO to pyrethroid-only nets [4–7, 19, 20], and supports current WHO recommendations favoring prioritization of pyrethroid-PBO nets over pyrethroid-only nets in areas with confirmed pyrethroid resistance [8]. While Vector Guard® induced higher mosquito mortality than Royal Sentry®, Olyset® Plus performed worse, with mortality rates even lower than those observed with Royal Sentry® 2.0. These differences likely reflect the type of pyrethroid used: alpha-cypermethrin in Vector Guard® versus permethrin in Olyset® Plus. Pre-exposure to PBO partially restored susceptibility to alpha-cypermethrin, raising mortality from 4% to 34%, but had little effect on permethrin, where mortality remained low (approx. 2%). This reduced synergism between PBO and permethrin, although not fully understood, is consistent with findings from previous studies conducted in Benin [13] and other settings in West Africa [21].

The non-inferiority analyses confirmed that Vector Guard® meets WHO criteria [11] for non-inferiority to Olyset® Plus in terms of both mortality and blood-feeding inhibition under unwashed, washed and pooled conditions. In fact, the performance of Vector Guard® exceeded that of Olyset® Plus across all endpoints, both in the hut trial and in the supplementary bioassays. Vector Guard® was associated with significantly higher mosquito mortality and lower blood-feeding rates, confirming not only non-inferiority but also superiority. The superior efficacy of Vector Guard compared to Olyset® Plus can also be explained by the afore-mentioned improved synergistic interaction between PBO and alpha-cypermethrin compared to permethrin. These findings suggest that pairing PBO with alpha-cypermethrin on ITNs (rather than with permethrin) can yield better entomological efficacy against metabolically resistant mosquito populations. Chemical analysis further substantiated these results. Vector Guard® exhibited high retention of both alpha-cypermethrin and PBO after 20 washes, with > 83% retention of PBO content and > 94% of alpha-cypermethrin content. This contrasted with Olyset® Plus, which retained only 40.2% of its PBO content and 69.6% of its permethrin. The higher wash-resistance index for PBO in Vector Guard® reflects its superior formulation and suggests greater durability of its insecticidal efficacy under household use.

Vector Guard® was added to the WHO list of prequalified vector control products [3] based on the results of this study and supporting evidence from a similar trial conducted against pyrethroid-resistant *Anopheles arabiensis* in Tanzania [22]. The Vector Guard® ITN is now commercialized under the brand name SafeNet® Plus after its ownership was transferred to Mainpol GmbH (Albershausen, Germany) in 2024 [23]. Given its demonstrated superiority over Olyset® Plus, Vector Guard® represents a strong alternative for malaria control programs seeking effective pyrethroid-PBO nets for use in areas with high-intensity pyrethroid resistance. Further operational research is warranted to assess its physical integrity and insecticidal durability under long-term community use.

## Conclusions

The Vector Guard® ITN demonstrated superior entomological efficacy and wash durability compared to both the Royal Sentry® 2.0 and Olyset® Plus ITNs, and fulfilled WHO non-inferiority criteria for mosquito mortality and blood-feeding inhibition. These findings support the inclusion of Vector Guard® as a strong candidate pyrethroid-PBO ITNs for malaria control in areas with high levels of pyrethroid resistance.

## Data Availability

Data supporting the main conclusions of this study are included in the manuscript.

## Abbreviations

AIRID: African Institute for Research in Infectious Diseases
cRCT: Cluster randomized controlled trial
CREC: Centre de Recherches Entomologiques de Cotonou
HDPE: High-density polyethylene
ITN: Insecticide-treated nets
*Kdr*: Knockdown resistance
LSHTM: London School of Hygiene & Tropical Medicine
NIM: Non-inferiority margin
P450: Cytochrome P450 monooxygenase
PAMVERC: Pan-African Malaria Vector Research Consortium
PBO: Piperonyl butoxide
PQT/VCP: Prequalification Unit Vector Control Product Assessment Team
